# Iris RESTful Server and IrisTileSource: An Iris implementation for existing OpenSeaDragon viewers

**DOI:** 10.1016/j.jpi.2025.100530

**Published:** 2025-11-17

**Authors:** Ryan Erik Landvater, Navin Kathawa, Mustafa Yousif, Ulysses Balis

**Affiliations:** University of Michigan Medical School, Department of Pathology, 2800 Plymouth Road, Ann Arbor, MI 48109-2800, USA

**Keywords:** Digital pathology, Whole-slide image, Iris, Iris File Extension, RESTful, OpenSeaDragon, TileSource

## Abstract

The Iris File Extension (IFE) is a low overhead performance-oriented whole-slide image (WSI) file format designed to improve the image rendering experience for pathologists and simplify image management for system administrators. However, static hypertext transfer protocol (HTTP) file servers cannot natively stream subregions of high-resolution image files, such as the IFE. The majority of contemporary WSI viewer systems are designed as browser-based web applications and leverage OpenSeaDragon as the tile-based rendering framework. These systems convert WSI files to Deep Zoom Images (DZI) for compatibility with simple static HTTP file servers. To address this limitation, we have developed the Iris RESTful Server, a low-overhead HTTP server with a RESTful application programming interface (API) that is natively compatible with the DICOMweb WADO-RS API. Written in C++ with Boost Beast HTTP and Asio networking libraries atop the public IFE libraries, the server offers both security and high performance. Testing shows that a single Raspberry Pi equivalent system can handle a peak of 5061 req./s (average 3883 req./s) with a median latency of 21 ms on a private (i.e., hospital) network. We also developed and merged a new OpenSeaDragon TileSource, compatible with the Iris RESTful API, into the next OpenSeaDragon release, enabling simple and immediate drop-in replacement of DZI images within WSI viewer stacks. Designed as a secure cross-origin resource sharing microservice, this architecture includes detailed deployment instructions for new or existing WSI workflows, and the public examples.restful.irisdigitalpathology.org subdomain is provided as a development tool to accelerate WSI web viewer development. All relevant Iris software is available under the open-source MIT software license.

## Introduction

**Fig. 1:**
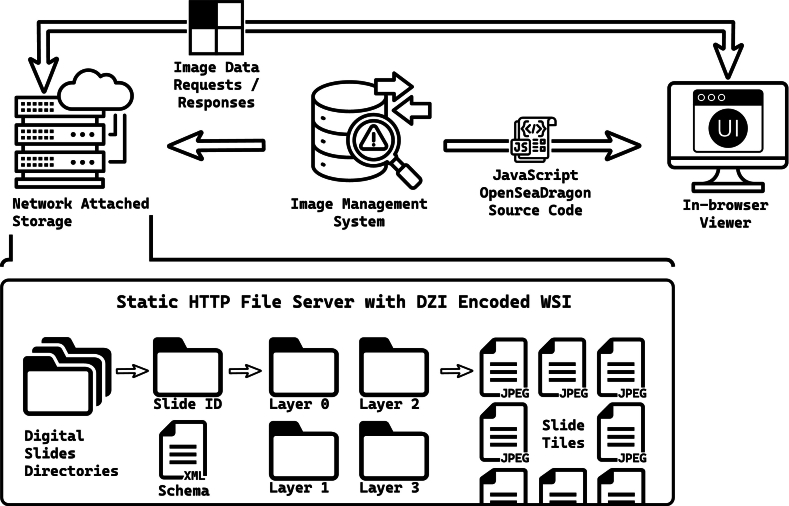
Current WSI digital pathology workflow. Digital slides are stored within a NAS system curated by an IMS such as a PACS. The IMS often provides both file curation with image retrieval and an WSV application by issuing OSD-based viewer source code to a browser on the pathologist client machine. The OSD WSV application requests regions of digital slide based on the current view port magnification and location on the slide. OSD viewer applications historically use DZI for native static HTTP file server compatibility and are stored as nested directories of individual image files, each encoding a single tile.

Digital pathology workflows, specifically whole-slide image (WSI) viewer systems, are being rapidly incorporated into a growing number of clinical practices in both academic and community pathology. These systems, broadly, involve a remote WSI file repository in the form of network-attached storage (NAS) and a whole-slide viewer (WSV) application that retrieves a portion of the image data from the NAS via a file server instance. The WSV then renders the image portion for pathologist’s viewing. Viewer systems are generally considered to be one of two types: (1) locally installed applications on the client (pathologist) workstation^1^^,^^2^ or (2) browser-based web applications,^3^^,^^4^ which are loaded in the form of HyperText Markup Language (HTML) and JavaScript (JS) source code from a server and run within the browser sandbox. Many image management system (IMS) solutions, such as hospitals' picture archiving and communication systems (PACSs), are responsible for curating the collection of digital slides within the NAS and have historically involved monolithic server deployments^5^ that include browser-based WSV applications (the second variety of the two described above). The general schema for this type of digital pathology workflow is shown in Fig. 1.

Advancements in the underlying rendering engines promise improved viewer responsiveness for both installed^1^ and browser-based WSV applications.^3^ The latter variety are aided by just-in-time (JIT) JS compilers for responsiveness closer to native code,^6^ but require simultaneous advancements in the methods we employ to access remote slide image data for these improvements to be realized. Current JS based viewers leverage OpenSeaDragon (OSD) as a super-resolution tiled rendering framework.^7^ OSD-based viewer systems historically use the Deep Zoom image (DZI) file format, which stores WSI files as a markup file—such as Extensible Markup Language (XML) or JavaScript Object Notation (JSON)—and a corresponding set of nested directories, each containing a single resolution layer within a multi-resolution image pyramid. Within each of these subdirectories are individual image files corresponding to a single rendered tile (Fig. 1, bottom pane). For more information on the OSD pyramidal rendering techniques for super-resolution images, we suggest the OSD rendering description by Shüffler et al.^8^

The DZI extension cleverly formats a high-resolution image in a structure natively compatible with any simple static HTTP file server. Unfortunately, this undesirably incorporates the operating system's file system as a critical internal component of the slide rendering software implementation. Additionally, this generates an expansive set of directories and subdirectories with which a file server's file system and the system administrators must contend. Finally, conversion to DZI from the original format, such as the Digital Imaging and Communications in Medicine (DICOM) format or a proprietary slide scanner format, can be onerous, time-consuming, and result in loss of clinical metadata. To address some of these issues, an OSD TileSource for digital pathology was developed to improve WSV performance by using the OpenSlide^9^ *DeepZoomGenerator* extension to convert to non-standard DZI formatted tiles with flexible tile sizes and layer magnifications instead of the sequential 2×-downsampling used in DZI conversion.^8^ However, it would be preferable to avoid the overhead of DZI conversion by issuing WSI tile data natively from a WSI file format.

We recently introduced the Iris File Extension (IFE) as a vendor-neutral open-source binary container file specification explicitly designed for performance-oriented WSI viewer systems that supports modern compression, a dynamic file structure, deep file validation routines, corruption recovery, and annotations.^10^ However, static HTTP file servers can only transmit complete files—or for large files like WSIs, must stream elements using specialized client JS code able to decode partial reads (‘Range Requests’ with HTTP 206 response). IFE, via the Iris Codec library, supports this type of advanced client-side WebAssembly module^10^ when files are hosted on static HTTP servers that can process range requests (like Amazon S3); however, such an implementation requires complex JS code and is still not as efficient as a server with native WSI streaming capabilities tailored to the file structure. A simple HTTP RESTful request structure is therefore far preferable. Herein, we introduce the Iris RESTful server, an open-source IFE compatible HTTP server implementation with both its own RESTful application programming interface (API) and compatibility with the DICOMweb's Web Access to DICOM Objects RESTful services (WADO-RS) API for ease of interoperability.

The Iris RESTful Server is written in C++ using the low-level and highly templated Boost Beast HTTP^11^ and Asio networking libraries, and is built on the open-source IFE libraries that we have made publicly available.^12^ The Iris RESTful Server was designed to be deployed as a modular and scalable containerized microservice accessible via a separate cross-origin resource sharing (CORS) domain to integrate with current IMS WSV applications. We also developed and merged a new OSD TileSource, the *IrisTileSource*, into the next OSD release so that IFE-encoded files can be immediately and seamlessly incorporated into existing digital pathology workflows. Incorporating *IrisTileSource* requires changing four lines of code within a viewer source (see Code listing 3 below). Finally, we make openly available the https://examples.restful.irisdigitalpathology.org/ subdomain, which we refer to as the **Iris RESTful Example domain**, as a fully Iris RESTful API compliant server with example IFE-encoded slides for community use as a tool to aid in streamlining the integration of IFE-encoded slides.

## Design

### Iris RESTful Server

**Fig. 2:**
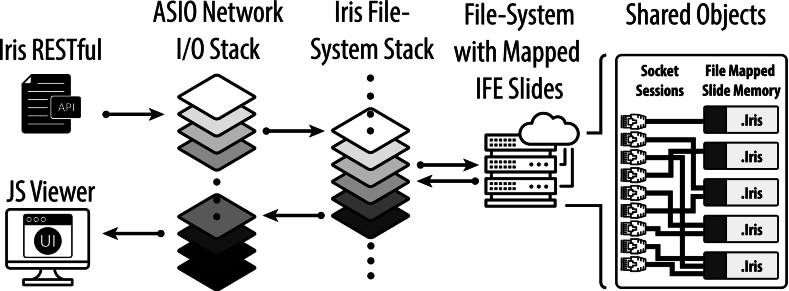
Iris RESTful server architecture. Isolated stacks execute the server requests on multiple independent threads in FIFO ordering. Asio Networking reactor threads are solely responsible for accepting packet requests and writing responses. The task of interpreting requests is forwarded to a separate Iris lockless file system stack and executed on a configurable number of separate independent threads. This reduces the pressure on the networking stack, limiting the network threads' scope to the execution of networking kernel calls in order to speed the draining of the network buffer and ensure high-reliability. File system kernel calls are isolated to the Iris stack, where slides are mapped into virtual memory space and shared between TCP socket sessions accessing the same slide resources.

The Iris RESTful server was designed as an extremely high-performance, lightweight, and secure dedicated slide-serving microservice. It is designed as a microservice to scale independently of the IMS with limited scope as it only returns slide tiles in response to Iris RESTful API requests—though the server may be optionally configured to perform static file services such as providing an OSD viewer web-application. The server comprises two independently operating stacks: (1) the networking stack and (2) the file system stack (Fig. 2). The server has security logic that parses API requests and discards requests that do not adhere strictly to the WADO-RS or Iris RESTful API (Code listings 1). When the server is configured as a static HTTP web server, it may also stream DZI image tiles for dual IFE and DZI compatibility; however, it will only return known file types and limit the file directory scope to ensure continued system security. Furthermore, the system automatically configures transport layer security (TLS) connections for end-to-end encryption when deployed with application load balancers to fulfill the in-transit Health Insurance Portability and Accountability Act (HIPAA) requirement.

**Listing 1:**
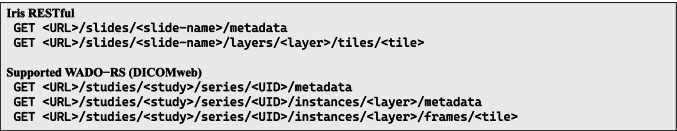
Iris RESTful API, acceptable GET commands. These URL target sequences retrieve slide metadata (in JSON form) and compressed slide tile bytestreams, respectively, using the Iris RESTful (*top*) and WADO-RS (*bottom*) APIs.

As mentioned above, the system has two core stacks (Fig. 2). Each stack is composed of a FIFO queue with multiple independent computer threads that add to and remove from the queue in a concurrent lockless manner. The division of the server implementation into two separate stacks uncouples networking calls from file system calls. This avoids prolonged computations or tasks from slowing down the server responsiveness to network requests; thus, one or more prolonged requests are isolated to a limited number of threads and passed over by the rest of the server. The Asio networking stack is solely dedicated to clearing the network buffer as quickly as possible to avoid dropping packets, thus increasing the server reliability and throughput.

The server is built on top of the public IFE (de)serialization library.^10^ When a client issues a new HTTP request, an Asio networking thread performs TCP connection procedures, executes a TLS/secure socket layer (SSL) handshake, and establishes a new secure TCP socket session. After validating the request format, the thread passes the request to the file system FIFO queue for interpretation. A file system thread looks into the server's active IFE slide handles and, if the slide is not presently open, validates the slide metadata against the IFE specification. It then creates a new IFE slide handle if the requested IFE file is present and has passed the IFE validation. In this way, multiple client sockets may benefit from shared IFE memory-mapped resources (Fig. 2, right-side). IFE slide handles are reference-counted server objects; they persist so long as at least one TCP socket remains open with a client viewer. When the last client socket referencing an IFE handle closes, the slide is unmapped from virtual memory space. During a request and after slide information is abstracted from the IFE slide handle, the slide data are serialized into a HTTP response and passed back to the Asio networking stack for transmission to the WSI viewer.

### OSD IrisTileSource

The *IrisTileSource*, a subclass derived from the OSD TileSource class, is designed to work seamlessly with the Iris RESTful Server API. The metadata and retrieve-tile overloaded functions are the main mechanisms by which the *IrisTileSource* class interfaces with a server instance via the Iris RESTful API (Code listings 1). The slide metadata is retrieved and parsed, and the *IrisTileSource* configures the OSD viewer instance by iterating over each layer tiles-extent and scale parameters. Viewer configuration is automatic. Subsequent tile requests follow a similar structure to DZI calls, wherein the tile location in *x* and *y* coordinates of layer *l* are converted into a URL-target sequence with a layer tile index *t* following a raster pattern according to the following eq. (1):

(1) $$t=y*\mathrm{tile}s_{l,x}+x$$

where $\mathrm{tile}s_{l,x}$ is the total number of tiles in the *x* dimension of layer *l*. A tile URL-target is thus generated per tile requests with the following structure:

**Table:**
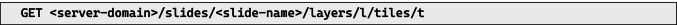

## Implementation

### Iris RESTful Server

**Fig. 3:**
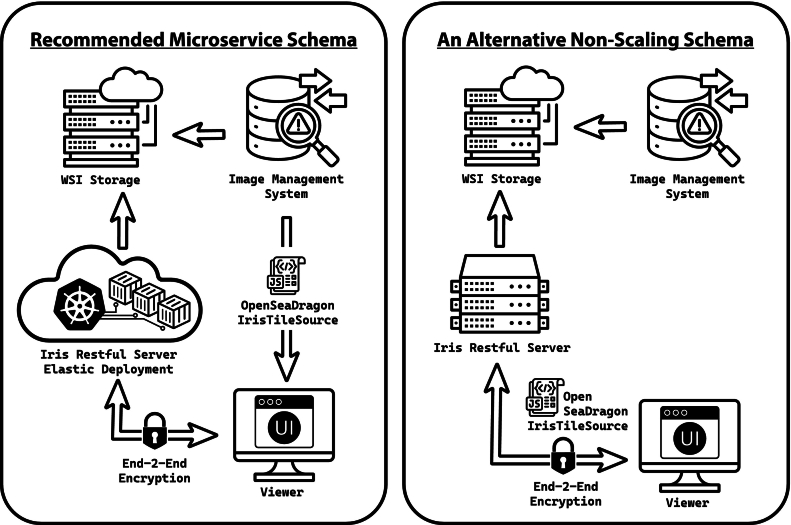
Iris RESTful within digital pathology workflows. (*left*) Our suggested architecture separates the IMS and WSV application from a scalable RESTful instance on a container orchestrator like Kubernetes (or AWS Elastic Container Service/Google Run). In this design, Iris RESTful is a CORS domain solely responsible for issuing slide tiles from the NAS. (*right*) In a different implementation, Iris RESTful is hosted on a server without container orchestration (but may be in or outside of a Docker container) with static HTTP server capabilities enabled—allowing it to issue the OSD-based WSV application directly. These are two opposite example architectures; however, there are many other viable workflow designs.

The server can be implemented as either a simple runtime executable or within a containerized environment. The server responds to both the Iris RESTful API and WADO-RS API, shown in Code listing 1. The full source code for the RESTful server with build configuration files are available on our Github Repository and can be compiled for any architecture; however, we recommend deployment as a elastic/scalable container service using the official Docker container images we host on our Github Container Repository. Please refer to the Data Availability section for more information on acquiring the source code and binary distributions. The proposed digital pathology workflow architectures are illustrated in Fig. 3, with our suggested scalable container architecture on the left. A simplified single instance server with mixed Iris RESTful and static HTTP file serving capabilities—which allows an Iris RESTful server to independently host a WSV application—is illustrated on the right. We will describe the steps for implementing the server below, starting with a basic example and expanding to the more advanced configuration.

In the most basic implementation, a server instance can be launched in a single line of bash code using a Docker run command (Code listing 2). Alternatively, the service can be run outside of a containerized instance by downloading one of the precompiled release binaries from the Github Iris-Restful-Server Releases page and configuring the server using the arguments in Code listing 2. When run in a container, the server is automatically configured with default arguments; however, these may be over-ridden using the Docker conventional methods for defining CMD parameters (see Docker documentation). A slide directory containing the IFE-encoded digital slide files is a required argument (Code listing 2, *argument −d/–dir*). To activate the static HTTP file serving capabilities, a document root directory may be specified (Code listing 2, *argument −r/–root*). It should be noted that Iris will prohibit clients from downloading IFE files even in this mode; therefore, you may safely contain slide files in the same directory as DZI or other permitted files for static file service without risking client IFE file download access. If Iris is configured for static file serving and is providing the WSV application, the CORS (cross-domain security policy specified with the ‘-o/–origin’ argument) can be disabled because the Iris RESTful is both the domain for the WSV application and the source of the slide tiles. This description corresponds with the right pane of Fig. 3.

**Listing 2:**
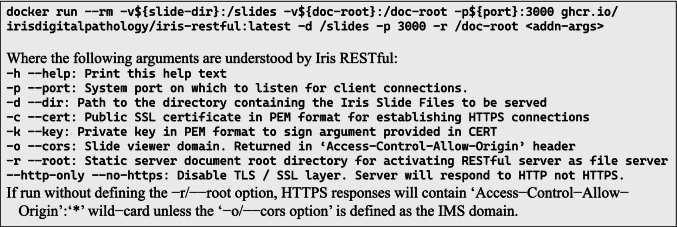
An example Docker deployment statement to begin running an Iris RESTful instance. A directory containing the IFE-encoded slide files must be mounted to the container. In Docker convention, arguments for the containerized application may follow the Docker image name and these optional arguments for Iris RESTful are shown below. For a more detailed explanation, visit the Iris-RESTful-Server Github Repository.

In a more scalable configuration, a cloud cluster service can be deployed using a container orchestrator system such as Kubernetes or Kubernetes-derived services such as those by Amazon Web Service (Elastic Container Service) or Google Cloud (Cloud Run). This design delegates the container scaling to a separate hypervisor system, and is more appropriate for large-scale implementations, such as institutions with dedicated hospital information technology offices familiar with scalable container orchestration services. Although static file serving capabilities can be enabled in this configuration (this is the architecture of examples.restful.irisdigitalpathology.org), for this type of production workflow, we recommend using the server as a CORS domain, where the IMS/PACS service (such as SECTRA AB or Roche) provides the WSV application from their PACS server instance and assigns the Iris-RESTful implementation as the OSD ‘serverUrl’ (see OSD *IrisTileSource* implementation Section titled 'OSD IrisTileSource' below). When deployed in this configuration, the RESTful server automatically generates self-signed 2048-bit RSA certificates that it uses to coordinate a secure connection with the container orchestrator/load balancer to ensure end-to-end encryption, even within a hospital's secure network or a provider virtual private network, fulfilling the in-transient HIPAA encryption requirement.

Please refer to the online documentation within the Iris-RESTful-Server Github repository for a more thorough and up-to-date explanation of server deployment strategies.

### OSD IrisTileSource

The *IrisTileSource* can be implemented within existing viewers by substituting only four lines of JS code in the OSD viewer class declaration. Rather than providing an OSD viewer “tileSources” parameter with the path to DZI image file, you must configure the OSD viewer “tileSources” as the new derived *IrisTileSource* class and assign that class the “serverUrl” parameter with the Iris RESTful server domain as well as the IFE-encoded slide file name (excluding the ‘.iris’ extension). See example HTML in Code listings 3 for a simple functional example web-viewer application.

**Listing 3:**
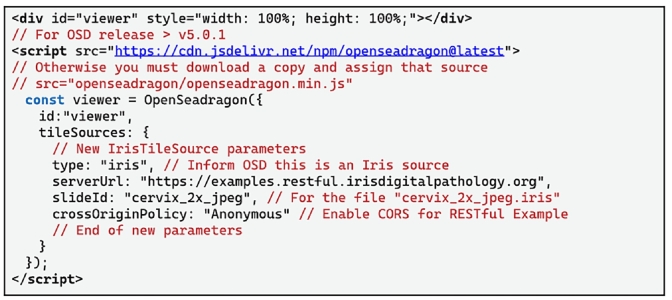
Simple HTML source that runs an OSD viewer using the *IrisTileSource* in only four lines of additional code using the latest version of OSD. The new *IrisTileSource* is assigned to the OSD viewer tileSources parameter and requires the new CORS serverUrl and slideID parameters. This example uses the Iris RESTful Example domain as the server and the cervix_2x_jpeg.iris example slide file. CORS is enabled using the Anonymous value.

### Error handling

The server is runtime hardened with numerous try-catch blocks that prevent runtime errors from escaping each stack. In the event of invalid requests or server-side errors, the server will terminate parsing only a single request and immediately respond with a series of HTTP error codes and response body narratives explaining the server error if such a typical error exists within the dictionary of known issues (such as failed validation or request range violations). The stack thread will then simply move on in the queue. The major response error categories are available within the *IrisRestfulTypes.hpp* class definitions. All error response descriptions are returned in the HTTP response body in plain-text form for ease of parsing.

## Performance results

### Materials and methods

The server distributions were built using GitHub Actions runners directly from the source repository using the latest versions of supported operating systems (GitHub Actions YAML compilation code is available at the repository website). Secondary builds were also performed using Google Cloud Build runners and evaluated for security insights. Vulnerability scans of these automated build artifacts from the source repository (Supply-chain Levels for Software Artifacts (SLSA),^13^ Build Level 3) could not identify any vulnerabilities in the resulting server deployment (vulnerabilities ranging from low to critical).

Illustrative performance testing was conducted for both a single-instance local network deployment (Fig. 3, *right*) and the public facing Iris RESTful Example domain in comparison to other well-established and publicly available WSI servers, specifically Path-Presenter and the National Cancer Institute (NCI) imaging data commons (IDC) Slim viewer.^6^ The slides were selected for similar imaging characteristics (JPEG-encoded 256 × 256 pixel tiles), which resulted in similar packet sizes. Iris Restful Example did not use a content delivery network (CDN); although we are unable to verify that CDN caching was not used by Slim or PathPresenter. Performance was evaluated using Locust,^15^ a widely accepted open-source and flexible load testing tool. Locust explicitly does not allow local data caching during a test. A ramped load test was chosen to allow for automatic instance scaling in the publicly facing WSI servers. Users were scaled up to 160 and 220 concurrent users for public WSI server and local network tests, respectively. Each virtual user issued a tile request at 1–2 ms intervals after receiving the prior response. Custom locust Python scripts were created for each target server, and tiles were randomly sampled from within image bounds. These scripts have been made available, along with the raw results of these tests (see Data Availability section). All tests were performed on a 2020 13-in. M1 MacBook Pro (Apple, Palo Alto, CA) with 8 GB of RAM running macOS 15.4, with Locust scripts executed using Python 3.11 in an Anaconda environment over a ≥200 Mbps wireless (Wifi) connection.

The single-instance local network deployment was hosted on Turing RTK1 (Turing Machines Inc., Cupertino, CA) 8-core Rockchip RK3588 system on module (SoM) circuit board with 8 GB of RAM running Ubuntu 24.04 within a Docker container and with a 1-gigabit Ethernet switch. The Turing RTK1 SoM is similar to the Raspberry Pi CM4 or the Jetson Orin Nx. The publicly facing Iris RESTful Example domain is hosted on AWS using an application load balancer in front of a Fargate elastic container service with 1 virtual CPU and 2 GB of RAM, and set to scale-out at either 70% CPU consumption or greater than 500 requests/s per instance (up to 100 USD/month with aggressive load testing). Both the local network deployment and Iris RESTful Example domain served IFE-formatted tiles using the Iris RESTful API. Path-Presenter served DZI-formatted tiles using a static HTTP server without an API. The NCI IDC repository served DICOM-formatted frames using the WADO-RS API.

### Server load testing

**Fig. 4:**
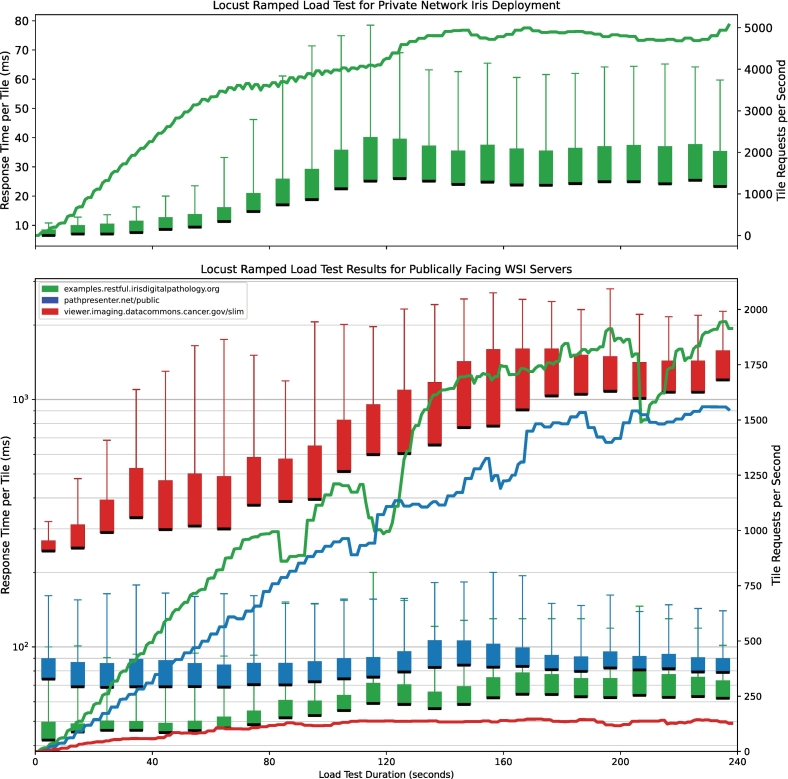
Ramped Locust server load testing performance. An ideal server shows low response time (boxes) while handling a high tile request rate (line trace). (*top*) Ramped load testing for a single Iris RESTful deployment on a local private network without container orchestration with 220 virtual users ramped at a rate of 1.8 usr/s. The half-box plots show median/50 percentile (bottom black line) to 75 percentile (top) and 95 percentile (whiskers) server response latency. The line trace shows total tile requests per second. (*bottom*) Comparisons of ramped load testing on publicly available WSI servers with 160 virtual users ramped at a rate a 1 user per second showing both tile response times (box-plots) and tile requests per second (line-trace) for Iris RESTful example server (green), Path-Presenter (blue), and NCI IDC Slim viewer (red). A log scale was used for tile response times in the bottom pane to aid in visualization due to substantially prolonged NCI IDC (DICOMweb) response times. (For interpretation of the references to color in this figure legend, the reader is referred to the web version of this article.)

Results from the local network deployment load tests (*n* = 932,269 requests) showed a median tile response time of 21 ms (range 2.7–450 ms) with an average tile request rate of 3883 requests/s over the entire test duration (Fig. 4, *top pane*). The public facing and scalable Iris RESTful Example domain load tests (*n* = 298,002 requests) showed a median response time of 59 ms (range 34.7 ms–2.59 s) with an average tile request rate of 1241 requests/s over the test duration (Fig. 4, *bottom pane, green*). Path-Presenter domain load tests (*n* = 237,102 requests) showed a median response time of 79 ms (range 56.8 ms–1.74 s) with an average tile request rate of 987 requests/s over the test duration (Fig. 4, *bottom pane, blue*). The Slim viewer domain load tests (*n* = 27,392 requests) showed a median response time of 650 ms (range 159.0 ms–23.7 s) with an average tile request rate of 114 requests/s over the test duration (Fig. 4, *bottom pane, red*). The complete results are shown in Table 1.

**Table 1:** Aggregate ramped Locust server load testing performance metrics. Iris Private Instance shows ramped load testing for a single Iris RESTful deployment on a local private network. Iris RESTful Example, Path-Presenter, and Slim show aggregate ramped load testing for publicly available WSI servers issuing IFE, DZI, and DICOM slides, respectively.

WSI server	Requests/s		Response times		
	Avg	Max	50%	75%	95%
Iris Private Instance	3883	5061	21 ms	33 ms	63 ms
Iris Restful Example	1241	1944	59 ms	71 ms	120 ms
Path-Presenter (DZI)	987	1560	79 ms	93 ms	160 ms
Slim (DICOMweb)	114	146	650 ms	1.2 s	2.3 s

## Discussion

We previously published on the rapid encoding speeds of IFE files from source WSI files such as DICOM due to the highly parallelized encoding capabilities afforded by the layer-agnostic tile ordering within the file specification.^10^ This aids in the value of the Iris RESTful server by allowing rapid conversion from source DICOM to IFE files, which may then be served to OSD viewers. The current versions of the Iris Codec IFE libraries can generate a derived pyramid file (with 2× or 4× downsampling) from a single highest-resolution DICOM or vendor format source within 1–2 mintues—or much faster if OpenSlide decoding^9^ is not needed. We continue to work on improving encoding systems and in future, releases will add stream encoding (a slide scanner API) to allow for simultaneous generation of archive DICOM and IFE files directly from the scanner to make IFE slides available to Iris RESTful in WSI workflows immediately. The IFE is in the public domain under a Creative Commons Attribution-No Derivative 4.0 license, and Iris Codec and IrisRESTful server implementations are available to the community under the MIT software license. The IrisTileSource is a part of the OSD repository and follows the OSD‘s BSD-3 license.

In this article, we performed stressful load testing on WSI systems to assess system durability and performance when these servers. This includes both expected daily loads early in the load test, as well as abnormally high volumes of requests. The early metrics better align with what we have previously observed with the Slim viewer of an approximately 200 ms median response time (Fig. 4, *left-side*) in our prior publication.^10^ The Iris RESTful Example domain response times of approximately 20–30 ms also align closely with what we experienced when using our example domain during the development of the *IrisTileSource* and example browser-based WSVs.

We are particularly happy with the server responsiveness on small and low-cost single-board computers such as the Turing RTK1 (which costs only $200 USD) and on 1 vCPU serverless AWS (Fargate) instances. The IFE implementations are designed with computationally trivial validations and low instruction set reads, while the Boost Beast library almost exclusively uses inline template functions to create a very lean and light server. The implications of this design include substantially lower hardware cost for WSI servers. Implementing a digital pathology workflow can be quite expensive. While the cost of the WSI server is just a fraction of the overall cost, it can represent an significant contribution. Leaner and more efficient systems benefit all potential health systems. We are particularly hopeful that with this performance on hardware carrying a smaller financial footprint, we hope that Iris RESTful can help to ensure globally accessible first-class digital pathology workflows for health systems regardless of the country or region's resource status.

## Conclusion

The Iris RESTful Server and OSD *IrisTileSource* allow for immediate and easy replacement of DZI images with IFE-encoded slide files within digital pathology WSI workflows. Prepackaged container images allow for server deployment in a scalable microservice architecture and require altering only four lines of viewer code to begin rendering with the new IrisTileSource within existing OSD viewers. Written in C++ with the highly templated Boost Beast library and on top of the IFE deserialization library, this is a low-overhead fast deployment that can issue slide tiles in 21 ms on average under a high request load of over 3883 tile requests per second, even when hosted on a small $200 USD compute module chipboard (the equivalent of a Raspberry-Pi CM4). We provide the Iris RESTful Example domain, a server that hosts example IFE-encoded slide files and responds natively to Iris RESTful API calls, for community use to evaluate HTTPS implementations with the Iris RESTful API and accelerate the development process. All relevant Iris implementations are available under the MIT software license.

## CRediT authorship contribution statement

RL designed and developed the Iris RESTful server, Iris RESTful API, performed the performance profiling, created the figures, wrote the introduction, performance results, and conclusion sections, and contributed to the design and implementation sections. NK authored the *IrisTileSource*, integrated it with OpenSeaDragon (including authoring additional documentation), and contributed to the design and implementation sections. MY provided guidance and insights on the structure of the Iris RESTful API and support of the WADO-RS API. UB provided guidance throughout the development. All authors reviewed and approved the final manuscript.

## Figures and Tables

**Table:** 

API	application programming interface.
CDN	content delivery network.
CORS	cross-origin resource sharing.
DICOM	Digital Imaging and Communications in Medicine.
DZI	Deep Zoom image.
FIFO	first-in-first-out.
HIPAA	Health Insurance Portability and Accountability Act.
HTML	HyperText Markup Language.
HTTP	Hypertext Transfer Protocol.
IDC	imaging data commons.
IFE	Iris File Extension.
IMS	image management system.
JIT	just-in-time.
JS	JavaScript.
JSON	JavaScript Object Notation.
NAS	network-attached storage.
NCI	National Cancer Institute.
OSD	OpenSeaDragon.
PACS	picture archiving and communication system.
SLSA	Supply-chain Levels for Software Artifacts.
SoM	system on module.
SSL	secure socket layer.
TCP	transmission control protocol.
TLS	transport layer security.
WADO-RS	Web Access to DICOM Objects RESTful services.
WSI	whole slide image.
WSV	whole slide viewer.
XML	Extensible Markup Language

## Data Availability

The official container images for the Iris RESTful server are available on the Github Container Repository (https://ghcr.io/irisdigitalpathology/iris-restful:latest). The JS source code for the *IrisTileSource* is available within the OSD Github Repository and can be evaluated more closely within OSD pull request #2759 (https://github.com/openseadragon/openseadragon/pull/2759). The complete source code for the Iris RESTful server is available at the official Iris Digital Pathology Github organization page (https://github.com/IrisDigitalPathology) with build instructions as well as precompiled binary artifacts with each release within the releases directory (https://github.com/IrisDigitalPathology/Iris-RESTful-Server/releases/latest). IFE-encoded slide files are available through the Iris RESTful Example domain (https://examples.restful.irisdigitalpathology.org) and at https://iris.example-slides.org. To generate IFE-encoded files, please refer to IFE online documentation (https://github.com/IrisDigitalPathology/Iris-Codec#implementations) or review the data availability statement (https://www.sciencedirect.com/science/article/pii/S2153353925000471#da0005) with the original IFE publication.^10^ The performance Locust test scripts and raw test results data (csv) are included as supplemental data with this publication.
